# Access to basic sanitation facilities reduces the prevalence of anaemia among women of reproductive age in sub-saharan Africa

**DOI:** 10.1186/s12889-023-16890-3

**Published:** 2023-10-13

**Authors:** Benamba Chanimbe, Abdul-Nasir Issah, Abraham Bangamsi Mahama, Daudi Yeboah, Mary Rachael Kpordoxah, Nura Shehu, Ngozi Mabel Chukwu, Michael Boah

**Affiliations:** 1 Department for Programmes Effectiveness, World Vision International, Accra, Ghana; 2 Department of Health Services, Planning, Management, and Economics, School of Public Health, University for Development Studies, Policy, Tamale, Ghana; 3 United Nations Children’s Fund (UNICEF), Sokoto Field Office, Sokoto, Nigeria; 4 Department of Epidemiology, Biostatistics, and Disease Control, School of Public Health, University for Development Studies, Tamale, Ghana; 5 Department of Global and International Health, School of Public Health, University for Development Studies, Tamale, Ghana; 6 United Nations Children’s Fund (UNICEF), Maiduguri Field Office, Nigeria

**Keywords:** Sanitation, Anaemia, Reproductive women, Determinants, Sub-saharan Africa

## Abstract

**Background:**

The prevalence (≈ 30%) of anaemia among women of reproductive age in Sub-Saharan Africa (SSA) is a significant concern. Additionally, less than half of households in the region have access to basic sanitation facilities, raising questions about the potential role of poor sanitation in increasing anaemia prevalence. To address this, we examined the relationship between access to basic sanitation facilities and the prevalence of anaemia among women of reproductive age in SSA.

**Methods:**

The study analysed cross-sectional household-level Demographic and Health Survey data from selected SSA countries. A total of 100,861 pregnant and non-pregnant women aged 15 to 49 from 27 countries were analysed. Access to basic sanitation and haemoglobin (Hb) levels were classified using WHO and UNICEF standards. To examine the link between access to basic sanitation facilities and the prevalence of anaemia, a multilevel regression analysis was conducted, which adjusted for country fixed-effects to ensure that the findings were not biassed by variations in country-level factors.

**Results:**

Nearly 37% (95% CI: 36.4, 37.9) of households had access to basic sanitation facilities, and 41% (95% CI: 40.8, 42.1) of women had Hb levels that indicated anaemia. Women with access to basic sanitation had a lower risk of anaemia than those without access (AOR = 0.95; 95% CI: 0.93, 0.98, *p* < 0.01). Factors, including maternal age, education, marital status, breastfeeding, health insurance enrollment, and wealth group, were also associated with anaemia prevalence.

**Conclusions:**

Anaemia is a severe public health problem among women of reproductive age across all 27 SSA countries analysed, with nearly four in ten being affected. Access to basic sanitation facilities was associated with a reduced anaemia risk. However, only slightly over a third of households had access to such facilities. Further research is required to examine the underlying mechanisms and inform effective interventions.

## Background

Anaemia is a global public health concern that affects millions of people, particularly in low- and middle-income countries (LMICs). The World Health Organization (WHO) defines anaemia as a condition in which the number of red blood cells or the amount of haemoglobin in the blood is insufficient to meet the body’s physiological needs [1]. Anaemia is a multifactorial disease that can be caused by nutrition-specific factors, non-nutritional factors, or a combination of both. Iron deficiency is the most prevalent of the recognised causes of anaemia, but other factors such as parasitic infections, genetic disorders, and chronic diseases can also contribute to its development [2, 3]. The global prevalence of anaemia among women of reproductive age is a major concern, as it has serious implications for maternal and child health. Women who are anaemic during pregnancy are at an increased risk of complications such as preterm delivery, miscarriage, stillbirth, low birth weight, and maternal mortality [4–6]. In addition, anaemia can lead to reduced physical work capacity, poor cognitive function, fatigue, and poor overall health and development [7–9].

Despite the significant burden of anaemia among women of reproductive age, progress towards achieving the global nutrition target of halving anaemia prevalence among women of reproductive age by 2030 has been slow, especially in sub-Saharan Africa (SSA). Recent estimates indicate that approximately 30% of women aged 15–49 worldwide were anaemic in 2019, and the relative drop in frequency since 2000 has been insufficient to reach the aim of halving anaemia prevalence among women of reproductive age by 2030 [10]. In some countries in western and central Africa (Mali, Benin, Nigeria, Senegal, Burkina Faso, Gabon, and Côte d’Ivoire), anaemia prevalence exceeded 50% in 2019. Iron interventions, such as fortification and iron supplementation, are the most commonly used strategies to control anaemia [1]. However, these interventions alone are insufficient to reduce the anaemia burden, especially in countries with high infection rates, such as SSA. Therefore, nutrition-sensitive strategies such as infection control are at least as important as iron therapies in these contexts to reduce anaemia [11].

Infection with soil-transmitted helminths (STHs) is one of the causes of anaemia globally, with hookworm being the most common species of STHs [12]. The distribution of STHs is widespread in tropical and subtropical areas, affecting over 1.5 billion people globally, with the highest incidence rates in SSA, the Americas, China, and East Asia. Being the most common species of STHs in SSA, hookworms have the potential to impact the epidemiology of anaemia in the region [13–15]. The risk of STHs increases with the practice of open defecation [14, 16]. Furthermore, population-based studies have established a connection between open defecation and haemoglobin (Hb) levels in both children and adults [17, 18]. However, the majority of existing policies implemented to manage STH infections primarily focus on controlling the associated morbidity. This is achieved through the administration of periodic preventive chemotherapy to people residing in endemic areas who are at risk of infection [19, 20]. While the implementation of preventive chemotherapy has been effective in reducing morbidity from helminth infections, the benefits are only short-term, as reinfection typically occurs rapidly after treatment [21].

Systematic reviews and meta-analyses have revealed that enhanced sanitation could make a substantial contribution to a long-term decline in the occurrence of STH infections in communities [22–24]. Globally, the percentage of people with access to at least basic sanitation facilities increased from 73 to 78% between 2015 and 2020, as reported by the WHO and United Nations Children’s Fund (UNICEF) Joint Monitoring Programme (JMP) for Water Supply, Sanitation, and Hygiene (WASH) [25]. However, in the African Union, only 42% of the population had access to basic sanitation services in 2020, with approximately 780 million people lacking these facilities [26].

The current inadequate literature on the association between sanitation access and anaemia prevalence in reproductive women in SSA raises the question of whether it is a contributing factor to the high occurrence of anaemia in the region. Nonetheless, there exists supporting evidence demonstrating an association between sanitation indicators and anaemia in other geographical locations. On a global scale, a study demonstrated a lower prevalence of anaemia among women residing in communities with complete sanitation coverage, highlighting a potential link between improved sanitation and reduced anaemia rates [27]. Furthermore, a national study of anaemia among reproductive women from South and Southeast Asian countries also reported an increased risk of anaemia for women lacking access to toilet facilities [28]. These aforementioned studies come from outside SSA and have not specifically provided empirical evidence on the association between sanitation and anaemia in the SSA context. We acknowledge that a study conducted in South Africa found notable disparities in Hb levels among women who had access to improved sanitation facilities compared to those who did not have such access. Nevertheless, it is important to note that evidence from a study conducted in a single country within SSA may lack generalizability to the entire region. This highlights the need for additional research to explore the potential impact of basic sanitation access on reducing the prevalence of anaemia in SSA.

In this context, the present study attempts to establish whether the prevalence of anaemia among women of reproductive age in SSA is associated with household access to at least basic sanitation facilities. The study utilises data from the most recent Demographic and Health Surveys (DHSs) conducted in twenty-seven countries in the SSA. The study hypothesises that inadequate access to basic sanitation facilities may be a contributing factor to the high prevalence of anaemia among women of reproductive age in SSA.

## Methods

### Conceptual framework

Upon thorough review of the literature, we have postulated two plausible pathways that may explain how sanitation could potentially impact the prevalence of anaemia among women of reproductive age. The first pathway is through the transmission of intestinal parasites, while the second pathway operates through the mechanism of environmental enteropathy. It is possible that these two pathways could function independently or in conjunction with each other, as both mechanisms are consistent with the detrimental effects of poor sanitation on human health. The prevalence of STHs has been linked with open defecation in various studies [14, 16, 29]. Hookworms, for instance, are a common STH that can cause anaemia. These parasites invade and attach themselves to the mucosa and submucosa of the small intestine, leading to significant blood and iron losses. Depending on the underlying iron status and the presence of other risk factors, such as poor nutritional reserves and iron deficiency, anaemia can occur [2].

The second pathway, environmental enteropathy, is characterised by polymicrobial infection with a wide range of enteropathogens, which are primarily transmitted via faecal-oral routes. Enteropathogens can cause inflammation, leakiness, and a reduction in surface area in the intestines, leading to deficiencies of micronutrients required for the production of haemoglobin, including vitamin B12, zinc, folic acid, and iron [30]. Both pathways provide a plausible explanation for the linkage between poor sanitation and the high prevalence of anaemia among women of reproductive age in SSA, highlighting the importance of improving access to basic sanitation facilities in the region.

### Data and sources

The present study utilises nationally representative cross-sectional household-level data derived from the DHS program in selected SSA countries. The demographic and health surveys are extensive in scale, providing all-encompassing data on a wide range of topics, including household characteristics, health status, and access to basic services such as sanitation facilities. The surveys implement a two-stage sampling technique, with the first stage comprising the selection of clusters of households using probability proportionate to the estimated population and the second stage encompassing the random selection of households within the chosen clusters.

Information was obtained from respondents’ self-reports, and the questionnaires used were standardised, adapted for different settings, and pre-tested to ensure comparability between countries. Water and sanitation data are collected at the household level in the DHS using core questions developed by the WHO and UNICEF [31]. Furthermore, the Biomarker Questionnaire collects information for each eligible household member on anthropometric measurements and levels of haemoglobin and records information about samples for biomarker testing. Typically, the collection of blood specimens for anaemia testing occurs in half of the selected households, which comprise women aged 15–49 who voluntarily consented to be tested. The standard approach for haemoglobin testing in population-based surveys using the HemoCue system has been explained elsewhere [32].

The present study used data from the most recent DHS conducted in each respective country. Specifically, only one survey was used per country. There were 27 countries and 157,022 observations with valid information on Hb levels. To ensure the absence of missing data on the variables included in the study, observations with missing data on exposure variables were discarded. Ultimately, 100,861 pregnant and non-pregnant women aged 15–49 were retained for analysis. The surveys were conducted between 2006 and 2020.

### Dependent variable

The dependent variable in the study is a categorical variable called anaemia status. The variable was determined based on the adjusted Hb levels, accounting for altitude and smoking status, which were then transformed into a categorical variable using the WHO Hb cutoffs for diagnosing anaemia among pregnant and non-pregnant women (15 years of age and older). Specifically, anaemia was defined as Hb levels less than 110 g/dl and 120 g/dl among pregnant and non-pregnant women, respectively, in accordance with the WHO criteria [33]. The severity of anaemia was also classified based on Hb measurements, with mild, moderate, and severe anaemia defined using different ranges of Hb levels for pregnant and non-pregnant women. For pregnant women, mild, moderate, and severe anaemia were defined as Hb measurements between 100 g/l and 109 g/l, 70 g/l -79 g/l, and less than 70 g/l, respectively. For non-pregnant women, mild, moderate, and severe anaemia were defined as Hb levels between 110 g/l and 119 g/l, 80 g/l − 109 g/l, and less than 80 g/l, respectively, according to the same WHO criteria.

### Main independent variable

The primary explanatory variable of interest in this study is household access to basic sanitation facilities. We operationalized this variable as the use of improved toilet facilities that are not shared with other households, in accordance with the definition provided by the WHO/UNICEF Joint Monitoring Programme for Water Supply, Sanitation, and Hygiene. These improved toilet facilities include flush or pour flush toilets connected to a piped sewer system, septic tank, or pit latrine, ventilated improved pit (VIP) latrines, pit latrines with slabs, and composting toilets, as per the most recent definition provided by the World Health Organization and the United Nations Children’s Fund [25]. Data on the type of toilet facility used by household members and whether the facility was shared with other households were collected through the demographic and health survey questionnaires.

### Covariates

It is important to acknowledge that a multitude of factors could potentially influence the occurrence of anaemia in women and therefore confound any potential association between sanitation and anaemia in the current study. To account for potential confounding factors that could influence the relationship between sanitation and anaemia in women, we incorporated a comprehensive set of individual, household, and contextual-level covariates. These factors include maternal age, the highest level of education, marital status, parity, pregnancy status, health insurance coverage, exposure to different types of media (print, audio, and audiovisual), sex of the household head, wealth status, and place of residence. The selection of covariates was informed by a review of previous literature [34–38], as well as the availability of these variables in the DHS dataset.

### Statistical analysis

The data analysis for this study was conducted using Stata/IC 15.0 (StataCorp LLC, College Station, USA) and involved both descriptive and multilevel inferential statistics. For each country and the entire study population, the percentage of households with access to basic sanitation facilities and the prevalence of anaemia were calculated. A bivariate analysis was conducted to compare the prevalence of anaemia among women across exposure and covariate variables, and variables with p-values less than 0.05 were included in the binary multilevel regression. The first model examined the variation in anaemia prevalence between clusters, while the second model investigated the association between access to basic sanitation and anaemia prevalence. The third model adjusted for individual-level factors, and the final model included household-level factors. All models were adjusted for country -fixed effects to ensure that the findings were not biassed by variations in country-level factors. Only statistically significant factors were included in subsequent models, and complex survey design and sample weights were taken into account using the “svy” command in Stata. Adjusted odds ratios (AORs) and corresponding 95% confidence intervals (CIs) were used to present the results of the multilevel logistic regression analysis, and the fit of the models was evaluated using the Akaike information criterion (AIC).

### Ethical considerations in the current study

For this study, we utilised DHS data that is accessible to the public. Prior to conducting the research, we received authorization from the DHS program through ICF International. The DHS survey protocol, which involves the collection of biomarkers, undergoes review and approval by both the implementing country’s ethics review committee and the Institutional Review Board of ICF International. All participants provided informed consent, and we used de-identified datasets.

## Results

### Percentage of households with access to at least basic sanitation facilities in the countries studied

Table 1 displays the percentage of households that have access to basic sanitation facilities. The findings indicate that 37.1% (95% CI: 36.4, 37.9) of households have access to at least basic sanitation facilities. Sierra Leone had the lowest access to sanitation facilities at 16.0% (95% CI: 13.9, 18.4), while Lesotho had the highest at 65.7% (95% CI: 59.7, 68.3).

**Table 1:** Percentage of households with access to basic sanitation facilities in 27 sub-Saharan African countries

Country	Survey year	Weighted number of women	Weighted percent	Percent of households with access to basic sanitation	
				Percent	95% CI
Benin	2017-18	2,812	2.8	23.3	[20.0,26.9]
Burkina	2010	2,287	2.3	37.8	[33.8,42.0]
Burundi	2016-17	6,530	6.5	43.6	[40.8,46.4]
Cameroon	2018	4,642	4.6	38.7	[34.8,42.7]
Cote D’Ivoire	2011-12	2,031	2.0	25.6	[21.8,29.7]
DR Congo	2017	7,682	7.6	20.3	[17.0,24.1]
Eswatini	2006-07	1,946	1.9	64.4	[60.0,68.6]
Gambia	2019-20	3,644	3.6	46.0	[41.7,50.3]
Ghana	2014	1,136	1.1	24.0	[19.5,29.1]
Guinea	2018	3,238	3.2	25.0	[22.3,27.9]
Lesotho	2014	1,016	1.0	65.7	[59.5,71.5]
Liberia	2019-20	1,561	1.6	29.9	[25.0,35.3]
Madagascar	2008-9	2,967	2.9	33.3	[29.5,37.4]
Malawi	2015-16	5,299	5.3	52.4	[49.8,55.1]
Mali	2018	4,454	4.4	33.4	[30.3,36.6]
Mozambique	2015	6,298	6.2	31.3	[29.0,33.7]
Namibia	2013	940	0.9	58.1	[52.8,63.3]
Niger	2017	1,499	1.5	31.1	[26.7,35.9]
Nigeria	2018	9,842	9.8	37.8	[35.3,40.3]
Rwanda	2019-20	3,971	3.9	58.2	[55.5,60.8]
Sao Tome and Principe	2008-09	620	0.6	45.0	[38.1,52.2]
Sierra Leone	2019	3,620	3.6	16.0	[13.9,18.4]
South Africa	2016	1,191	1.2	54.0	[48.4,59.5]
Tanzania	2015-16	8,111	8.0	46.2	[43.7,48.7]
Togo	2013-14	1,350	1.3	22.7	[19.1,26.8]
Zambia	2018	8,172	8.1	30.7	[28.1,33.6]
Zimbabwe	2015-16	4,004	4.0	42.9	[39.6,46.2]
Total	2006-20	100,861	100.0	37.1	[36.4,37.9]

### Prevalence and distribution of anaemia among women aged 15–49 years in the 27 sub-Saharan African countries

**Fig. 1:**
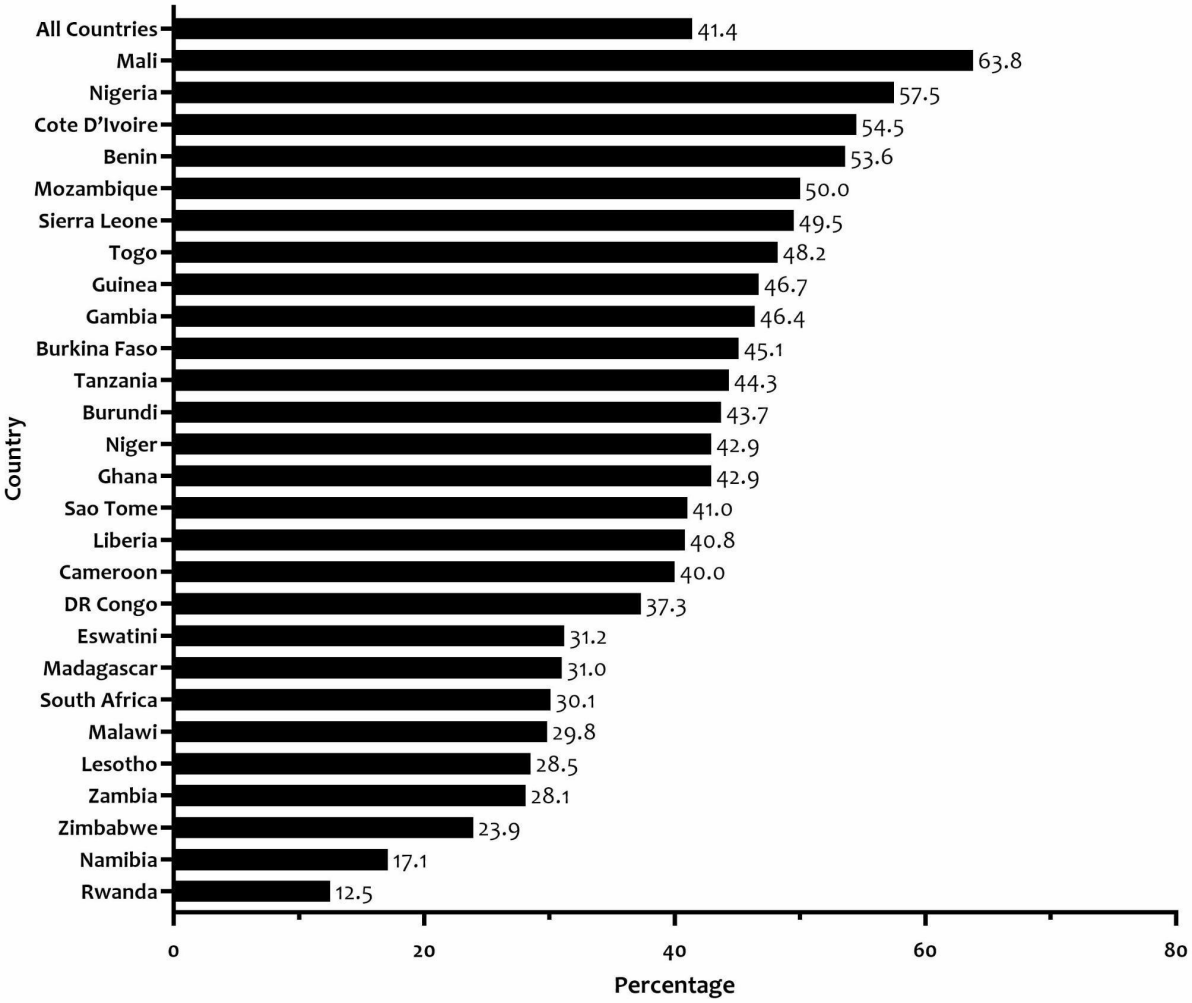
Prevalence of anaemia among women aged 15–49 years in 27 sub-Saharan African countries (N = 100,861)

The mean Hb for both pregnant and non-pregnant women was 120.9 ± 17.2 d/dl (122.1 ± 16.7 g/dl for non-pregnant women and 109.1 ± 16.6 g/dl for pregnant women). Of all pregnant and non-pregnant women, 49.7% (95% CI: 48.1, 51.3) and 40.6% (95% CI: 39.9, 41.2), respectively, had Hb levels indicative of anaemia. Overall, 41.4% (95% CI: 40.8, 42.1) of the total sample had anaemia, ranging from 12.5% (95% CI: 10.9, 14.2) in Rwanda to 63.8% (95% CI: 61.0, 66.5) in Mali (Fig. 1). Mild anaemia was observed in 53.2%, moderate anaemia in 43.5%, and severe anaemia in 3.3% of the population.

The prevalence of anaemia was significantly lower among women residing in households with access to at least basic sanitation facilities (38.6%) compared to those without access (43.1%). Various maternal sociodemographic, economic, and media-related factors were associated with a lower prevalence of anaemia, including age group 25–34 years (40.8%), not being currently in a union (39.0%), not being pregnant (40.5%), not breastfeeding (40.8%), and having two liveborns (39.6%). Furthermore, the prevalence of anaemia was lower among women who read newspapers or magazines (35.5%), listened to the radio (40.5%), had health insurance (28.7%), lived in female-headed households (39.7%), and were from the richest households (38.8%) (Table 2).

**Table 2:** Distribution of pooled prevalence of anaemia across the explanatory variables in the study (N = 100,861)

Variable	Anaemia prevalence	*P*-value
	Percent [95% CI]	
Access to basic sanitation		< 0.001
No	43.1[42.3,43.9]	
Yes	38.6[37.7,39.5]	
Age group (years)		0.016
15–24	41.6[40.7,42.6]	
25–34	40.8[40.0,41.6]	
35–49	42.5[41.5,43.6]	
Educational level		< 0.001
No education	50.7[49.7,51.8]	
Primary	38.1[37.2,39.0]	
Secondary	36.6[32.7,37.6]	
Higher	35.1[32.7,37.6]	
Current union status		< 0.001
Not in a union	39.0[37.8,40.2]	
Currently in a union	41.8[41.2,42.5]	
Currently pregnant		< 0.001
No	40.5[39.9,41.2]	
Yes	49.9[48.3,51.5]	
Currently breastfeeding		0.015
No	40.8[40.0,41.5]	
Yes	42.0[41.2,42.8]	
Parity		< 0.001
1	39.7[38.7,40.7]	
2	39.6[38.5,40.8]	
3	40.1[38.9, 41.4]	
4+	43.3[42.4,44.2]	
Reads newspaper		< 0.001
No	42.8[42.1,43.5]	
Yes	35.5[34.4,36.6]	
Listens to the radio		< 0.001
No	42.9[41.9,43.8]	
Yes	40.5[39.7,41.2]	
Watches television		0.792
No	41.4[40.6,42.2]	
Yes	41.5[40.7,42.4]	
Covered by health insurance		< 0.001
No	42.7[42.0,43.3]	
Yes	28.7[27.1,30.3]	
Sex of household head		< 0.001
Male	41.9[41.2,42.6]	
Female	39.7[38.7,40.8]	
Wealth		< 0.001
Poorest	43.6[42.1,45.0]	
Poorer	43.2[41.9,44.5]	
Middle	42.3[41.1,43.4]	
Richer	40.4[39.3,41.6]	
Richest	38.8[37.7,39.8]	
Place of residence		0.087
Urban	40.7[39.8,41.7]	
Rural	41.8[41.0,42.7]	

### Mixed-effects regression analysis of the association between household access to basic sanitation facilities and anaemia among women in 27 sub-Saharan African countries

Table 3 presents the results of both fixed and random-effects regression analyses that examined the relationship between household access to basic sanitation facilities and anaemia prevalence among women aged 15–49 years. The regression analyses, both univariate and adjusted, found that access to basic sanitation facilities was associated with a lower likelihood of anaemia among women. Specifically, the accepted model showed that women whose households had access to basic sanitation facilities had a 5% reduced likelihood of anaemia (AOR = 0.95, 95% CI: 0.93, 0.98) compared to those from households without access to basic sanitation facilities.

**Table 3:** Fixed and random-effects multilevel regression analysis of the association between access to basic sanitation facilities and anaemia among women in 27 sub-Saharan African countries

Variable	Model 1	Model 2	Model 3	Model 4
		OR [95% CI]	AOR [95% CI]	AOR [95% CI]
Access to basic sanitation				
No		1.00		1.00
Yes		0.82^***^[0.80,0.84]	0.94^***^[0.91,0.97]	0.95^**^[0.93,0.98]
Individual-level factors				
Age group (years)				
15–24				1.00
25–34			0.97[0.93,1.01]	0.97^*^[0.94,1.00]
35–49			1.06^*^[1.00,1.11]	1.06^**^[1.01,1.10]
Educational level				
No education				1.00
Primary			0.86^***^[0.83,0.89]	0.88^***^[0.84,0.91]
Secondary			0.77^***^[0.74,0.80]	0.82^***^[0.78,0.85]
Higher			0.67^***^[0.61,0.73]	0.73^***^[0.67,0.80]
Current union status				
Not in a union				1.00
Currently in a union			0.84^***^[0.80,0.87]	0.85^***^[0.81,0.88]
Currently pregnant				
No				1.00
Yes			1.45^***^[1.38,1.52]	1.44^***^[1.37,1.50]
Currently breastfeeding				
No				1.00
Yes			1.08^***^[1.05,1.11]	1.07^***^[1.03,1.10]
Parity				
1			1.00	
2			0.985[0.94,1.03]	
3			0.969[0.92,1.02]	
4+			0.987[0.94,1.04]	
Reads newspaper				
No			1.00	
Yes			1.015[0.98,1.06]	
Listens to the radio				
No			1.00	1.00
Yes			0.93^***^[0.90,0.96]	0.95^***^[0.92,0.98]
Covered by health insurance				
No			1.00	1.00
Yes			0.90^***^[0.85,0.96]	0.92^**^[0.86,0.98]
Household-level factors				
Sex of household head				
Male				1.00
Female				1.03[0.99,1.07]
Wealth				
Poorest				1.00
Poorer				0.96[0.92,1.01]
Middle				0.93^**^[0.88,0.97]
Richer				0.87^***^[0.83,0.92]
Richest				0.83^***^[0.79,0.88]
Random-effects results				
PSU variance [95% CI]	0.18[0.15,0.21]	0.18[0.15,0.21]	0.11[0.09,014]	0.11[0.09,0.13]
ICC	0.05	0.05	0.03	0.03
Wald χ^2^	Reference	205.55	6063.35	6111.25
Model fit				
Log- likelihood	-68799.051	-68695.807	--65349.12	-65319.68
AIC	137602.1	137397.6	130784.2	130727.4
Number of observations	100,861	100,861	100,861	100,861
Number of clusters	1327	1327	1327	1327

Exponentiated coefficients; 95% confidence intervals in brackets. Analyses adjusted for country fixed-effects

^*^p < 0.05, ^**^p < 0.01, ^***^p < 0.001

**Fig. 2:**
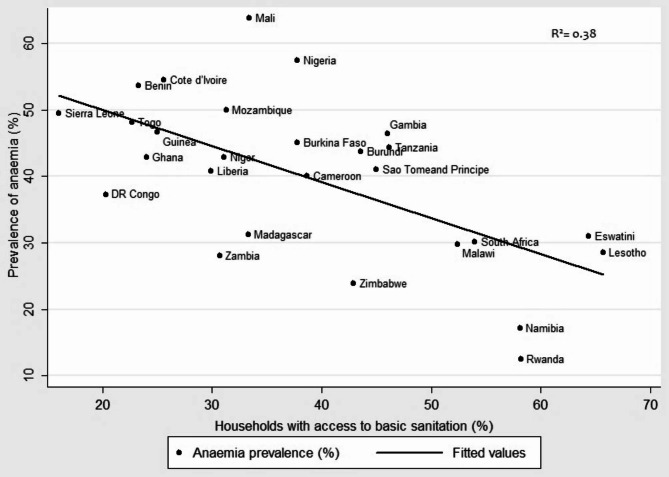
Univariate association between sanitation access and anaemia among women aged 15–49 in 27 sub-Saharan African countries

Furthermore, a scatter plot was used to examine the association between household access to basic sanitation and anaemia prevalence among women. The plot showed that access to basic sanitation was a strong determinant of anaemia in some countries but a weak determinant in others. Univariate linear regression explained 38.0% of the variation in anaemia prevalence (Fig. 2). Lesotho, with the highest proportion of households with access to basic sanitation (65.7%), had an anaemia prevalence of 28.5%, while Rwanda, with 58.2% of households having access to basic sanitation, had a prevalence of 12.5%. Similarly, Mali had the highest prevalence of anaemia in the study (63.8%), but only 33.4% of households had access to basic sanitation, while Sierra Leone had an almost 50% prevalence of anaemia, but only 16% of households had access to basic sanitation facilities.

Additionally, the final regression model showed that several factors were significantly associated with the likelihood of anaemia among women aged 15–49 years. Women with primary (AOR = 0.88, 95% CI: 0.84, 0.91), secondary (AOR = 0.82, 95% CI: 0.78, 0.85) or higher levels of education (AOR = 0.73, 95% CI: 0.67, 0.80) had a lower likelihood of anaemia compared to women with no formal education. Women who were currently in a union (AOR = 0.85, 95% CI: 0.81, 0.88), listened to the radio (AOR = 0.95, 95% CI: 0.92, 0.98), and were covered by health insurance (AOR = 0.92, 95% CI: 0.86, 0.98) also had a lower likelihood of anaemia. Women from households in the middle (AOR = 0.93, 95% CI: 0.88, 0.97), richer (AOR = 0.87, 95% CI: 0.83, 0.91), and richest wealth index (AOR = 0.83, 95% CI: 0.79, 0.88) had a lower likelihood of anaemia compared to the poorest wealth index group. On the other hand, women in the age group of 35–49 years (AOR = 1.06, 95% CI: 1.01, 1.10), those currently pregnant (AOR = 1.44, 95% CI: 1.37, 1.50), and those currently breastfeeding (AOR = 1.07, 95% CI: 1.03, 1.10) had a higher likelihood of anaemia compared to their counterparts.

### Random-effects results

Model 1 shows that 5% of the total variation in anaemia prevalence is attributable to variation between clusters. However, in Model 3, the variation between clusters is only 3%. Additionally, Model 3 has the lowest AIC (130727.4) and log-likelihood (-65319.7) values and the highest Wald chi-square test value (6111.3) compared to the other models, indicating that it is the best-fitting model for the multilevel regression analysis (Table 3).

## Discussion

Anaemia is a significant public health problem affecting women of reproductive age in SSA. In this study, we examined the association between the accessibility of basic sanitation facilities in households and the likelihood of anaemia among women of reproductive age residing in the SSA. Our findings showed that access to basic sanitation facilities in SSA is limited, with only about one in three households having access, and significant disparities exist among countries, with the highest percentage observed in Lesotho and the lowest in Sierra Leone. Additionally, roughly four in ten women, regardless of their pregnancy status, showed haemoglobin levels indicative of anaemia, with the highest incidence in Mali and the lowest in Rwanda. The prevalence of anaemia was found to be higher among pregnant women than non-pregnant women, likely due to the augmented demand for iron by the foetus, placenta, and maternal blood volume during pregnancy [8, 39]. A comprehensive examination of nationally representative DHS data from earlier studies revealed comparable trends characterised by notable discrepancies in access to basic sanitation facilities and the prevalence of anaemia among women in their reproductive years [27, 40]. The prevalence of anaemia was also found to be significantly higher among pregnant women in comparison to non-pregnant women.

According to the WHO’s categorization of the public health significance of anaemia based on prevalence estimated from Hb levels, none of the 27 SSA countries studied fell within the normal prevalence range of less than 4.99% [33]. From our data, anaemia among women of reproductive age is a moderate public health problem in SSA. However, the severity of the problem varied by country, with seventeen countries (Burkina Faso, Benin, Burundi, Cameroon, Cote D’Ivoire, Gambia, Ghana, Guinea, Liberia, Mali, Mozambique, Niger, Nigeria, Sao Tome and Principe, Tanzania, Togo, and Sierra Leone) classified as having a severe (40% or higher) public health problem, eight countries (Dr Congo, Eswatini, Lesotho, Madagascar, Malawi, South Africa, Zambia, and Zimbabwe) as having a moderate (20.0 − 39.9%) public health problem, and two countries (Namibia and Rwanda) as having a mild (5.0 − 19.9%) public health problem.

The variation in the prevalence of anaemia between countries may be attributed to differences in socioeconomic status and underlying causes, including the burden of infectious diseases [12, 41]. At the global and country levels, the burden of anaemia decreased with increasing socioeconomic development in 2019 [41]. Furthermore, reductions in malaria incidence have been identified as a contributing factor to the low prevalence of anaemia in some SSA countries, particularly during 2005–2015 when there were rapid increases in the use of insecticide-treated nets and reductions in malaria incidence [42]. However, malaria incidence remains higher in West and Central Africa than in other regions, which may have contributed to the high prevalence of anaemia in these sub-regions.

The primary focus of the current study was to establish a link between household access to basic sanitation facilities and the prevalence of anaemia among women of reproductive age. Our comprehensive analysis revealed compelling evidence of a reduced likelihood of anaemia among women of reproductive age who have access to basic sanitation facilities in their households. This effect persisted even after accounting for potential confounding factors such as individual and household-level characteristics as well as the influence of the country. These findings confirm our hypothesis and suggest that basic sanitation facilities could potentially play a protective role in mitigating the occurrence of anaemia among women and highlight the urgent need for increased efforts to improve access to basic sanitation facilities, which are essential to addressing the problem of anaemia in SSA. One plausible explanation for the lower anaemia risk among women with access to basic sanitation facilities could be attributed to the reduced levels of parasitism, such as hookworm infections, and the impact of environmental enteropathy. Indeed, prior studies have linked infections with STHs, particularly hookworm, to open defecation practices [14, 16, 29].

A further exploration of the relationship using simple linear regression, however, demonstrated that the effect of access to basic sanitation on the prevalence of anaemia may not be the same in all the countries examined. Specifically, it appears that access to basic sanitation facilities may play a more prominent role in the epidemiology of anaemia in some countries than in others and that sociodemographic and economic factors may be more influential in certain contexts [34, 35, 38]. Nevertheless, we observed a general pattern whereby in countries where anaemia is a severe public health issue, less than half of the population had access to basic sanitation facilities, with most of these countries located in the central and western regions of SSA. Collectively, our results indicate that, while enhancing access to basic sanitation facilities could be a significant approach to mitigating the prevalence of anaemia among women of reproductive age in SSA, it is likely to be more effective when combined with other interventions that address the underlying causes of anaemia, including improving access to nutritious food, reducing the burden of infectious diseases, and improving overall socioeconomic status [41, 43, 44].

The final regression model found that other factors, including having formal education, being in a union, enrolling in health insurance, listening to the radio, and belonging to a wealthy group, were all associated with a reduced prevalence of anaemia among reproductive women in the current study. These findings are consistent with findings from country-specific studies [34, 36, 38]. Additionally, the mechanisms through which these factors operate have been extensively discussed in the literature. For instance, improved socioeconomic status resulting from education, wealth, and media exposure has been linked to a reduced incidence of malaria infection, a significant risk factor for anaemia [44]. This positive effect can be attributed to the enhanced access to resources, better job opportunities, and increased knowledge of social issues, financial planning, and entrepreneurship that education, wealth, and media exposure provide. On the other hand, the study found that pregnant or breastfeeding women had a higher risk of anaemia, which is consistent with previous research by Ali et al. [37], Kibret et al. [34], and Nankinga & Aguta [36]. In alignment with the current study’s findings, a Ugandan study associated older maternal age with high levels of anaemia among women aged 35–49 [36]. In contrast, a study in Ethiopia reported that anaemia was less prevalent among older women than young women [34].

### Policy implications

The present investigation found unacceptable levels of anaemia among women of reproductive age in the examined population, a condition that has been linked to poor access to basic sanitation facilities within households. The policy implications of these findings are significant. There is a need for increased investment in improving access to basic sanitation facilities, particularly in central and western SSA countries where access is particularly low. This could involve initiatives such as improving infrastructure for safe water and sanitation, promoting hygiene education, and providing financial support to households to build and maintain recommended sanitation facilities. Nonetheless, to achieve optimal effectiveness, this strategy should be implemented in tandem with other interventions that target the root causes of anaemia, such as improving access to nutritionally adequate diets, lessening the burden of infectious diseases, and enhancing the socioeconomic status of individuals [41, 43, 44]. Overall, our study underscores the need for a comprehensive and coordinated approach to reducing the burden of anaemia in SSA that addresses the underlying determinants of the condition, including access to basic sanitation facilities and socioeconomic factors. Improving the overall socioeconomic status of populations in SSA could involve initiatives such as improving access to education, promoting economic growth, and implementing social protection programmes [45].

### Strengths and Limitations

The study’s use of nationally representative data from the DHS program is a significant strength, allowing for the generalisation of findings to women of reproductive age in SSA. However, the examination of the relationship between basic sanitation and anaemia was only done for the pooled dataset and not for individual countries, which may have concealed any significant or opposite associations in smaller countries. The cross-sectional design also limits the ability to make causal inferences. Hence, the findings should be interpreted in light of these strengths and limitations. Despite these constraints, our investigation underscores the elevated burden of anaemia among women of childbearing age in SSA and emphasises the crucial role of basic sanitation in mitigating this burden.

## Conclusions

Nearly four in ten pregnant and non-pregnant women of reproductive age in the 27 countries included in the current study were anaemic, with anaemia being a severe public health problem in about two-thirds of the countries. This study has shown that among women of reproductive age, coming from a household with access to basic sanitation facilities lowers the likelihood of being anaemic. However, slightly over a third of households have access to basic sanitation facilities. Furthermore, the strength of the association may vary between countries, and other factors, including sociodemographic and economic factors, may also contribute to the epidemiology of anaemia. Nonetheless, the results highlight the importance of improving access to basic sanitation facilities as a potential strategy for reducing the burden of anaemia in SSA. Further research is needed to explore the mechanisms underlying this relationship.

## Data Availability

The DHS data used in the study are openly available, and interested researchers can obtain the datasets directly from the DHS program website (www.dhsprogram.com) and replicate the study following the procedures outlined in the methods section of the manuscript.

## Abbreviations

AIC: Akaike information criterion
AORs: Adjusted odds ratios
CIs: confidence intervals
DHSs: Demographic and Health Surveys
JMP: Joint Monitoring Programme
Hb: haemoglobin
LMICs: low- and middle-income countries
STHs: soil-transmitted helminths
SSA: Sub-Saharan Africa
UNICEF: United Nations Children’s Fund
VIP: ventilated improved pit
WHO: World Health Organization
